# TMPyP binding evokes a complex, tunable nanomechanical response in DNA

**DOI:** 10.1093/nar/gkae560

**Published:** 2024-06-29

**Authors:** Balázs Kretzer, Levente Herényi, Gabriella Csík, Eszter Supala, Ádám Orosz, Hedvig Tordai, Bálint Kiss, Miklós Kellermayer

**Affiliations:** Department of Biophysics and Radiation Biology, Semmelweis University, Tűzoltó Str. 37-47, H1094 Budapest, Hungary; HUNREN-SE Biophysical Virology Group, Tűzoltó Str. 37-47, H1094 Budapest, Hungary; Department of Biophysics and Radiation Biology, Semmelweis University, Tűzoltó Str. 37-47, H1094 Budapest, Hungary; Department of Biophysics and Radiation Biology, Semmelweis University, Tűzoltó Str. 37-47, H1094 Budapest, Hungary; Department of Biophysics and Radiation Biology, Semmelweis University, Tűzoltó Str. 37-47, H1094 Budapest, Hungary; Department of Biophysics and Radiation Biology, Semmelweis University, Tűzoltó Str. 37-47, H1094 Budapest, Hungary; Department of Biophysics and Radiation Biology, Semmelweis University, Tűzoltó Str. 37-47, H1094 Budapest, Hungary; Department of Biophysics and Radiation Biology, Semmelweis University, Tűzoltó Str. 37-47, H1094 Budapest, Hungary; HUNREN-SE Biophysical Virology Group, Tűzoltó Str. 37-47, H1094 Budapest, Hungary; Department of Biophysics and Radiation Biology, Semmelweis University, Tűzoltó Str. 37-47, H1094 Budapest, Hungary; HUNREN-SE Biophysical Virology Group, Tűzoltó Str. 37-47, H1094 Budapest, Hungary

## Abstract

TMPyP is a porphyrin capable of DNA binding and used in photodynamic therapy and G-quadruplex stabilization. Despite its broad applications, TMPyP’s effect on DNA nanomechanics is unknown. Here we investigated, by manipulating λ-phage DNA with optical tweezers combined with microfluidics in equilibrium and perturbation kinetic experiments, how TMPyP influences DNA nanomechanics across wide ranges of TMPyP concentration (5–5120 nM), mechanical force (0–100 pN), NaCl concentration (0.01–1 M) and pulling rate (0.2–20 μm/s). Complex responses were recorded, for the analysis of which we introduced a simple mathematical model. TMPyP binding, which is a highly dynamic process, leads to dsDNA lengthening and softening. dsDNA stability increased at low (<10 nM) TMPyP concentrations, then decreased progressively upon increasing TMPyP concentration. Overstretch cooperativity decreased, due most likely to mechanical roadblocks of ssDNA-bound TMPyP. TMPyP binding increased ssDNA’s contour length. The addition of NaCl at high (1 M) concentration competed with the TMPyP-evoked nanomechanical changes. Because the largest amplitude of the changes is induced by the pharmacologically relevant TMPyP concentration range, this porphyrin derivative may be used to tune DNA’s structure and properties, hence control the wide array of biomolecular DNA-dependent processes including replication, transcription, condensation and repair.

## Graphical Abstract

**Graphical Abstract:**
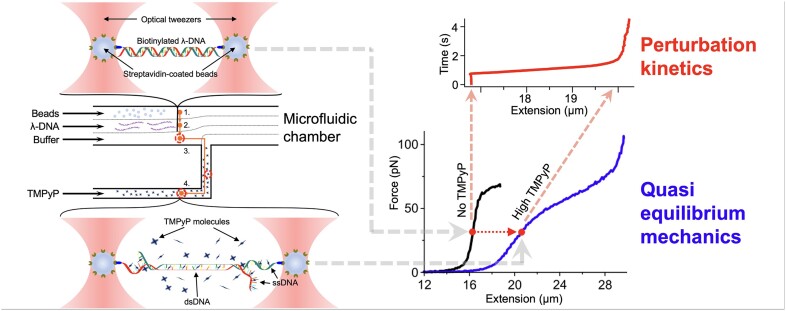

## Introduction

The advent and perfection of single-molecule methods in recent decades have led to unprecedented new insights into the mechanisms and properties of biomolecular processes, and pointed out that mechanical forces and molecular nanomechanics play a much more important role in controlling cellular and sub-cellular phenomena than earlier thought. DNA has particularly been in the focus of single-molecule experiments, as the molecule lends itself naturally to exploring the role of its axial, bending, twisting and strand-separation nanomechanics in DNA-associated processes (1–5). The combination of novel methodologies, for example, optical tweezers, microfluidics and high-resolution fluorescence microscopies, has paved the way towards understanding how DNA nanomechanics are influenced by external factors such as intercalators (6), binding proteins (7) or solute concentration gradients (8). Understanding DNA nanomechanics is key not only to uncover the mechanisms of the vast array of DNA-related intracellular processes, for instance, DNA replication and repair, chromatin condensation, gene transcription and regulation, but also in the development of novel, high-efficacy pharmaceuticals that specifically and sensitively influence these DNA-dependent processes (9,10).

DNA nanomechanics are influenced by DNA-binding molecules that interact with DNA in a variety of ways (9–13). Certain proteins and small molecules bind to the major or minor grooves of dsDNA, thereby altering its structure (14) and stability (10,15–20). Others may display electrostatic or allosteric interactions with DNA, but neither of them results in the disruption of genome continuity (10). Intercalators non-covalently insert their planar aromatic moieties between adjacent basepairs of dsDNA, thereby altering DNA’s structural and mechanical properties and perturbing enzymatic reactions crucial for cell proliferation and survival (6,10). Intercalation follows the rule of nearest-neighbor exclusion, hence adjacent basepairs are affected by every moiety intercalated (10,21). Intercalators typically lead to the stabilization, elongation and helix unwinding of dsDNA (10). Despite the pronounced structural and mechanical changes, intercalation is a reversible process, making intercalators promising candidates for a wide range of applications targeting DNA (6,10). By contrast, ssDNA-binding proteins and small molecules destabilize dsDNA (13,22,23). Altogether, DNA nanomechanics can be controlled by ligand binding *via* numerous synergistic and antagonistic mechanisms.

Porphyrins constitute an important and much investigated group of DNA-binding ligands, one of which, tetrakis(4-N-methyl)pyridyl-porphyrin (TMPyP), is the subject of the present paper. Porphyrins and their derivatives are widely known for their use in photodynamic tumor therapy (24–27). TMPyP, a cationic porphyrin, stands out with its strong DNA affinity and fluorescent properties, although because of its low quantum yield it is unlikely to be suitable for single-molecule imaging applications (28). TMPyP and its derivatives are also used as building blocks in functional assemblies (29–32). Cationic porphyrin derivatives have a broad antimicrobial effect (24,33–36), and TMPyP has specifically been investigated for its virus-inactivating properties (37,38). The interaction of TMPyP with G-quadruplexes sparked major interest, as TMPyP alters the mechanical properties of the G-quadruplex, thereby perturbing telomerase activity (39–41), raising the serious possibility that TMPyP and its derivatives may be successfully employed in cancer treatment (24,42). Prior studies have shown that TMPyP can intercalate between basepairs due to its planar structure, and it can also bind to the minor groove of the dsDNA (37,38,43). The preferred type of binding depends on TMPyP concentration, ionic strength and DNA sequence; however, the simultaneous presence of the different binding modes can be detected even at low TMPyP/basepair ratios (37,38). TMPyP is also capable of binding to ssDNA, and it catalyzes the formation of dsDNA (44,45). The binding reactions occur on the 10–500 ms time scale, and intercalation has been shown to be slower than groove binding (46). Despite the extensive investigation of TMPyP binding to DNA, its effect on DNA nanomechanics is still unknown.

In the present work, we carried out a comprehensive analysis of the effects of a wide range of TMPyP concentration, which includes the therapeutic concentration range, on the nanomechanical behavior, manifested in the force *versus* extension function, of DNA. Furthermore, we tested the effects of NaCl concentration and pulling rate on the TMPyP-dependent DNA nanomechanics. A complex array of nanomechanical response was measured, which we analyzed with a newly-developed empirical mathematical model that provided insight into the molecular mechanisms of the effects. Equilibrium and perturbation experiments allowed us to unveil thermodynamic and kinetic parameters of TMPyP-DNA binding and dissociation. Our results imply that DNA nanomechanics, hence important DNA-dependent processes such as replication, transcription, condensation and repair, may be finely tuned by an interplay between nanomolar TMPyP concentrations and piconewton forces.

## Materials and methods

### Samples and buffer solutions

**Figure 1.**
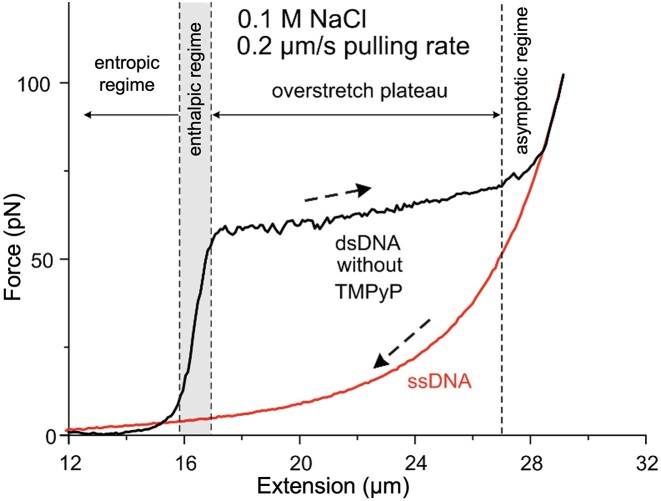
Canonical force versus extension curves (FECs) of dsDNA (black) and ssDNA (red). Raw experimental data are shown for a single molecule of dsDNA overstretched to be converted into ssDNA. The FECs are shown as reference for the subsequent mechanical experiments. To obtain the FEC of dsDNA, one of the strands of a single λ-phage DNA molecule was biotinylated on both the 3′ and 5′ ends and was extended with a constant rate (0.2 μm/s). The pulling was carried out in the absence of TMPyP, and in the presence of 0.1 M NaCl (20 mmol/l Tris–HCl, pH 7.4). At the end of the asymptotic part of the force trace we applied flow to wash off the complementary strand, and relaxed the remaining single-stranded DNA (ssDNA, red trace). Dashed arrows indicate the direction of data acquisition. The four characteristic nanomechanical regimes (entropic, enthalpic, overstretch plateau, asymptotic part) are separated by dashed vertical lines.

For the entire set of nanomechanical measurements we used λ-phage dsDNA biotinylated at its 3′-3′ ends (Lumicks, Amsterdam, The Netherlands). To generate the data in Figure 1, λ-phage dsDNA biotinylated on its 5′-3′ ends was used (Lumicks, Amsterdam, The Netherlands). In all experiments, DNA was diluted to a final concentration of 20 ng/ml. DNA was tethered between two 3.11-μm diameter streptavidin-coated polystyrene microbeads (Kisker Biotech, Steinfurt, Germany). TMPyP (Porphychem, Dijon, France) was used at different concentrations indicated in the figures. Tris–HCl buffer (20 mM Tris–HCl, pH 7.4) was used throughout the measurements. NaCl was added in different concentrations (0.01, 0.1 and 1 M). To inhibit the non-specific binding of positively charged TMPyP to the negatively charged glass surfaces, hence the alteration of the effective TMPyP concentration inside the flow cell, Tween-20 was added to the buffer at a final concentration of 0.01% (v/v). TMPyP concentration was measured by absorbance at 423 nm, by using a 4E UV-VIS absorption spectrophotometer (Varian, Inc., Palo Alto, CA).

### Stretching single DNA molecules

Experiments were performed by using a dual-trap optical tweezers instrument coupled with a multi-channel microfluidic system (Lumicks, C-Trap, Amsterdam, The Netherlands). Streptavidin-coated microbeads were captured with the optical traps, and single molecules of biotinylated λ-phage dsDNA were tethered between them. By moving one of the beads, the tethered DNA was stretched and the resulting force was recorded. Following the simultaneous measurement of force and inter-bead distance, force–extension curves (FECs) of the single DNA molecules were plotted. To minimize TMPyP concentration fluctuations resulting from non-specific adsorption to the internal surfaces of the microfluidic chamber, the flow cell was incubated with the respective buffer for 45 minutes prior to the measurements. To obtain the force curve of a single DNA molecule, first it was pulled in buffer without TMPyP (control measurement) at a constant, pre-adjusted pulling rate. Subsequently, the molecule was brought to the microfluidic channel containing TMPyP at the given concentration, and it was positioned far from the channel wall so that diffusion-driven transport processes would not influence the effective TMPyP concentration (6,8). Then, the pulling cycle was repeated with the same rate. To alleviate hydrodynamic perturbations on DNA, flow was halted during the pulling cycle. Three sets of measurement were performed, each at different NaCl concentrations (0.01, 0.1 and 1 M). In a single measurement series we gradually increased the TMPyP concentration from 5 nM to 5120 nM, which resulted in ten measurement sets for each series. In each measurement set, we collected several FECs at different pulling rates (0.2, 2, 20 μm/s). Thus, a multi-parametric dataset was recorded systematically with varying NaCl and TMPyP concentrations and pulling rates. For stretching ssDNA, a single λ-phage DNA molecule biotinylated on both the 3′ and 5′ ends was manipulated. After pulling the molecule beyond the overstretch transition the separated complementary strand was washed off.

### Perturbation kinetics on single dsDNA molecules

The kinetics of TMPyP binding to dsDNA were measured in force- and concentration-jump experiments. In force-jump experiments the TMPyP-dsDNA binding equilibrium, at either 5 or 80 nM TMPyP, was perturbed by rapid, stepwise increment in force, which was then held constant with a feedback loop (bandwidth 500 Hz). Force was increased in ∼5 pN steps, each requiring ∼30 ms, from 0 to 60 pN. Only length data collected after force stabilization were considered for extracting the *k_OBS_* of the binding reaction. During the force-jump experiments solution flow was completely halted with solenoid valves at the in- and outlets of the microfluidic chamber. In concentration-jump experiments the dsDNA molecule, held at constant force (10, 20, 30, 40 or 50 pN), was brought rapidly (within ∼250 ms) from 0 TMPyP to high TMPyP (5 or 80 nM) and back. 0 and high-TMPyP-concentration regions were maintained by rapid laminar flow (500 μm/s in the focal plane) in the microfluidic chamber, and moving the DNA molecule between the regions was achieved by moving the microscope stage holding the chamber. Length data collected after settling of the stage movement were considered for extracting the *k_OBS_* of the binding and dissociation reactions.

### AFM imaging of dsDNA

300-bp-long λ-phage dsDNA fragments were imaged in liquid at 25°C by using an Asylum Research Cypher ES atomic force microscope (Oxford Instruments, Abingdon, UK). Sample surfaces were scanned in tapping mode with BL-AC40TS (Olympus) cantilevers, resonated by photothermal excitation near the resonance frequency (∼20 kHz). Typical scan speeds were around 0.5 μm/s. Scanning resolution was 512 pixels/line for all images. 100 μl of DNA sample was dropped onto dried, poly-l-lysine (PLL)-covered mica surface. To measure the effects of TMPyP, dsDNA bound to the PLL surface was incubated with 250 nM TMPyP for 10 min. Image post-processing and analysis were performed within the AFM driving software (IgorPro, WaveMetrics, Portland, OR, USA). The contour length and end-to-end distance of individual DNA strands (*n* = 75) were measured for both control and TMPyP-treated samples by manually tracing along their axis. The ends of the DNA molecule were defined as the points at the half-maximal topographical height along the axial trace (see Figure 5B, C).

### Modeling the nanomechanical response of dsDNA

We developed an empirical mathematical model to fit the measured FECs and to understand which phases of dsDNA’s force response are most sensitive to a given experimental parameter. This model consists of three components: a sigmoid function (*f*_1_, entropic and enthalpic regimes), a linear function (*f*_2_, overstretch plateau) and a hyperbola (*f*_3_, asymptotic regime) (see Figure 3A):


(1) \begin{eqnarray*}{f_1}\left( x \right) = \frac{1}{{1 + exp\left( { - \frac{{x - {P_1}}}{{{P_2}}}} \right)}}\end{eqnarray*}

(2) \begin{eqnarray*}{f_2}\left( x \right) = {P_3}x + {P_4}\end{eqnarray*}

(3) \begin{eqnarray*}{f_3}\left( x \right) = \frac{{ - {P_6}}}{{\left( {x - {P_5}} \right)}}\end{eqnarray*}

where the fitting parameters are marked with *P_i_*. The meaning of the individual parameters is the following:

*P*_1_: position of the sigmoid curve along the x-axis; scales with the contour length of dsDNA.
*P*_2_: step width of the sigmoid curve; scales with the compliance of dsDNA in the enthalpic region.
*P*_3_: slope of the linear function; scales inversely with the cooperativity of the overstretch transition.
*P*_4_: y-axis intercept of the linear function; scales with the height of the overstretch plateau, hence with the stability of dsDNA.
*P*_5_: location of the asymptote of the hyperbola; scales with the maximal length of overstretched DNA (contour length of ssDNA).
*P*_6_: curvature of the hyperbola; related remotely to the bending rigidity of ssDNA.

Adding Equations (2) and (3) and multiplying by Equation (1) yields the following function:


(4) \begin{eqnarray*}{f_4}\left( x \right) = {f_1}\left( {{f_2} + {f_3}} \right)\end{eqnarray*}

which was used to fit all the measured FECs.

### Data analysis and visualization

**Figure 2.**
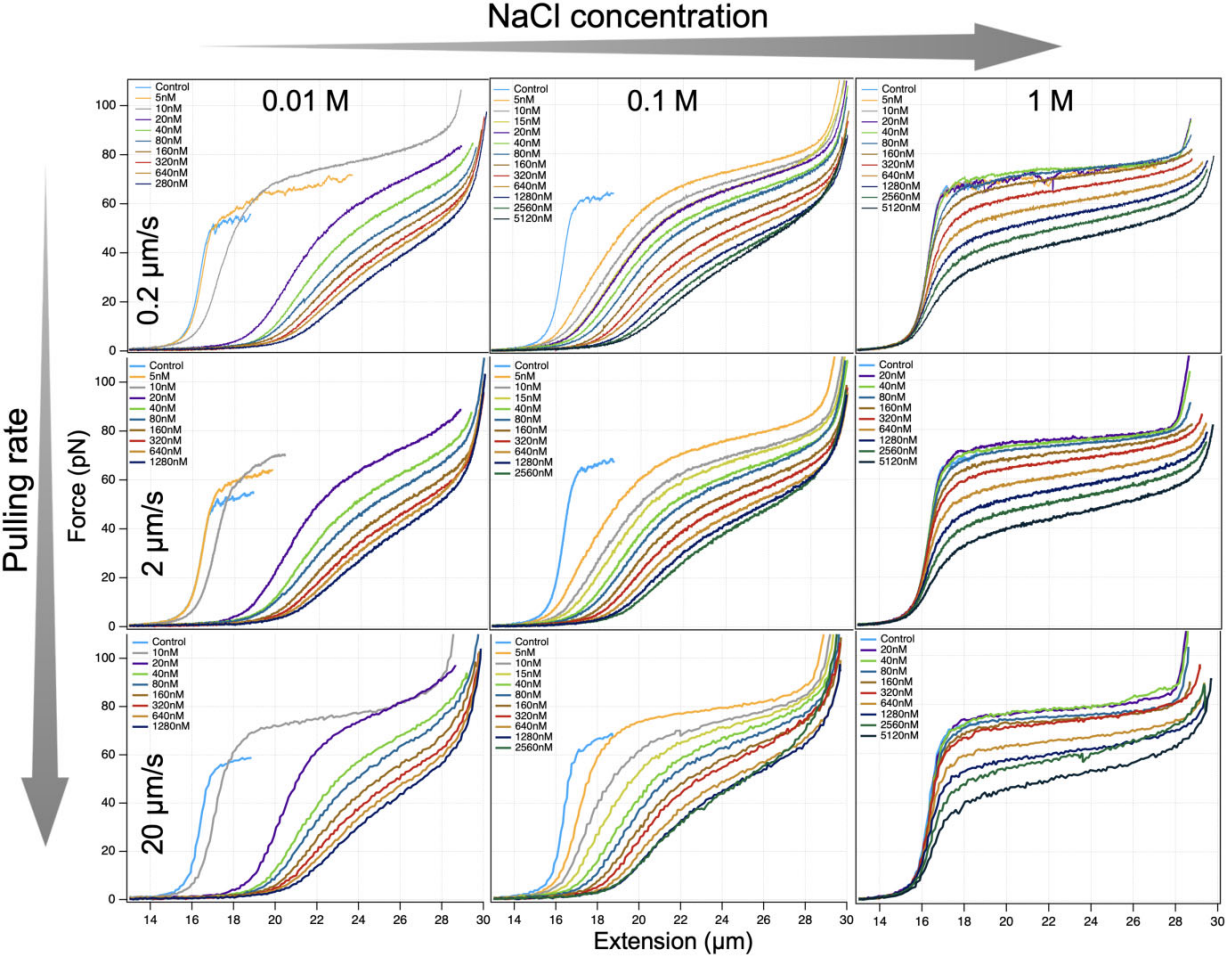
Effect of TMPyP concentration (indicated in the legend of each figure), pulling rate (indicated on the left side of each row) and NaCl concentration (indicated above each column) on the FEC of λ-phage DNA. Nine sets of TMPyP concentration-dependent FECs are shown in the pulling-rate and NaCl-concentration phase spaces. In the case of the 10 mM NaCl measurements, the maximum TMPyP concentration at which single dsDNA molecules could be reliably stretched was 1280 nM. Above this concentration only multimers of DNA molecules could be captured. These multimers likely form by enhanced chain-chain association screenable by increasing NaCl concentration.

Raw data from the optical tweezers experiments were converted and analyzed using Python 3.9′s matplotlib 3.3.4 library and the lumicks.pylake 0.7.2 package. Three independent measurements were done for each distinct setting of the complex parameter space (shown in Figure 2). The typical noise in the force data, estimated as the standard deviation (SD) during DNA overstretch transition, was only ±0.37 pN. Therefore, raw data are displayed throughout the paper, except for derived parameters where sample size and SD are indicated. Fitting Equation (4) to the data was carried out by using Origin (Northampton, Massachusetts, USA). Experimental force versus extension curves were fitted, by using IgorPro (version 9), with either the non-extensible (47)


(5) \begin{eqnarray*}\frac{{F{L_P}}}{{{k_B}T}} = \frac{z}{{{L_C}}} + \frac{1}{{4{{\left( {1 - z/{L_C}} \right)}^2}}} - \frac{1}{4}\end{eqnarray*}

or the extensible wormlike chain model (48,49)


(6) \begin{eqnarray*}\frac{{F{L_P}}}{{{k_B}T}} = \frac{z}{{{L_C}}} + \frac{1}{{4{{\left( {1 - z/{L_C} + F/\kappa } \right)}^2}}} - \frac{1}{4} - \frac{F}{\kappa }\end{eqnarray*}

where *F*, *L_P_*, *k_B_*, *T*, *z*, *L_C_* and *κ* are force, persistence length, Boltzmann's constant, absolute temperature, extension, contour length and stretch modulus, respectively. The apparent persistence length of surface-adsorbed dsDNA was calculated from the end-to-end distance (*R*) and contour length, measured in AFM images, by using the equation (50)


(7) \begin{eqnarray*}{L_P} = \frac{{\left\langle {{R^2}} \right\rangle }}{{4{L_C}}}\end{eqnarray*}

Equilibrium dsDNA length change (Δ*L*) *versus* force data obtained at different TMPyP concentrations ([*TMPyP*]) were fitted with the multi-site binding isotherm (6)


(8) \begin{eqnarray*}{\mathrm{\Delta }}L\left( F \right) = \frac{{{\mathrm{\Delta }}{x_{eq}}{N_{bp}}}}{n}{\left( {1 + \frac{{{e^{ - F{\mathrm{\Delta }}{x_{eq}}/{k_B}T}}}}{{n{K_0}\left[ {TMPyP} \right]}}} \right)^{ - 1}},\end{eqnarray*}

where Δ*x_eq_* is the equilibrium length change caused by the binding of a single TMPyP molecule, *N_bp_* is the full length of λ-phage DNA (48502 base pairs), *n* is the apparent TMPyP footprint (number of base pairs along DNA per TMPyP molecule) and *K*_0_ is the affinity constant at zero force. Force-dependent rate constants (*k_OBS_*) obtained in perturbation kinetics (force- or concentration-jump) experiments were fitted with the equation


(9) \begin{eqnarray*}{k_{OBS}}\left( F \right) = {k_0}{e^{F{\mathrm{\Delta }}x/{k_B}T}},\end{eqnarray*}

where *k*_0_ is the rate at zero force and Δ*x* is the apparent length change caused by the binding of a single TMPyP molecule. *k_ON_* and *k_OFF_* were estimated by fitting TMPyP-concentration-dependent *k*_0_ data with the equation (51)


(10) \begin{eqnarray*}{k_{OBS}} = {k_{ON}}\left[ {TMPyP} \right] + {k_{OFF}}.\end{eqnarray*}

In extreme experimental conditions, when the dsDNA molecule is switched rapidly between high and zero concentration of TMPyP, Equation (10) simplifies into *k_OBS_* = *k_ON_*[*TMPyP*] and *k_OBS_* = *k_OFF_*, for the high- and zero-concentration experiments, respectively. Statistical data analysis of AFM images was done in the program R (version 4.1.0). Data visualization was carried out in Origin, CorelDraw, Inkscape (version 0.92), KaleidaGraph (version 5.0.5), IgorPro (version 9) and R (version 4.1.0).

## Results

### Reference force–extension curve of DNA

To assess the effects of TMPyP on the nanomechanical behavior of DNA, we first measured a reference force–extension curve (FEC) (Figure 1). This typical FEC reveals the nanomechanical behavior of double-stranded (ds) and single-stranded (ss) λ-DNA without any DNA-binding molecule present. In the particular experiment shown in Figure 1, the dsDNA molecule was torsionally unconstrained and topologically open. We recorded a characteristic FEC that could be divided into four different regimes: (i) entropic regime, (ii) enthalpic regime, (iii) overstretch plateau and (iv) asymptotic regime. In the entropic regime the molecule is greatly extended by low (<∼10 pN) pulling forces which reduce configurational entropy, and the end-to-end distance of dsDNA approaches its contour length. In the enthalpic regime, a linear force response is observed, the slope of which is related to the stretch modulus (κ) of dsDNA. In the overstretch plateau, dsDNA is extended beyond its native length at the expense of several structural transitions (B–S transition, melting-bubble formation, strand unpeeling) that occur cooperatively within a narrow, ionic strength-dependent force range (52). The features (height, length) of the overstretch plateau reflect the stability of dsDNA, hence they are sensitive to the presence of intercalators and groove-binding molecules (6,9–13,19,53–56) or ssDNA-binding proteins (13,22,23). In the final stage of the stretch force curve the overstretch plateau is followed by an asymptotic regime, an elastic region where further elongation of DNA requires high forces. In this regime the two strands are held together by a few GC-rich regions (5). Since in this experiment DNA was biotinylated on the 3′ and 5′ ends of the same strand, washing the mechanically dissociated DNA strand away and relaxing the molecular system yielded the FEC of the remaining ssDNA, which was characterized as a wormlike chain (Figure 1). The obtained reference FEC allows us to uncover the mechanistic details behind the effects of TMPyP binding to DNA.

### Force-extension curves in the TMPyP, NaCl and pulling rate parameter space

We recorded dsDNA FECs across a wide range of TMPyP concentrations (doubling from 5 to 5120 nM) at three different NaCl concentrations (0.01, 0.1 and 1 M) and pulling rates (0.2, 2 and 20 μm/s), so that nine series of data were obtained. Figure 2 displays the entire dataset, with one FEC shown for a single experimental setting. Increasing TMPyP concentration led to drastic changes in the FEC so that all of the distinct regimes of the canonical FEC (Figure 1) were affected: the contour length of dsDNA increased, the slope in the enthalpic regime decreased, the height and slope of the overstretch transition decreased and increased, respectively, and the maximum contour length of the overstretched DNA increased. Upon increasing the pulling rate, we observed a slight recovery from the TMPyP effects (Figure 2, from top to bottom), suggesting that some of the changes are influenced by the thermodynamics and kinetics of the molecular system. Upon increasing the concentration of NaCl to 1 M, a significant recovery from the TMPyP effects was observed in the entropic and enthalpic regimes but not in the overstretch regime (Figure 2, from left to right), indicating that ionic strength has a differential effect on the DNA-TMPyP interaction.

### Extraction of nanomechanical details with an empirical mathematical model

**Figure 3.**
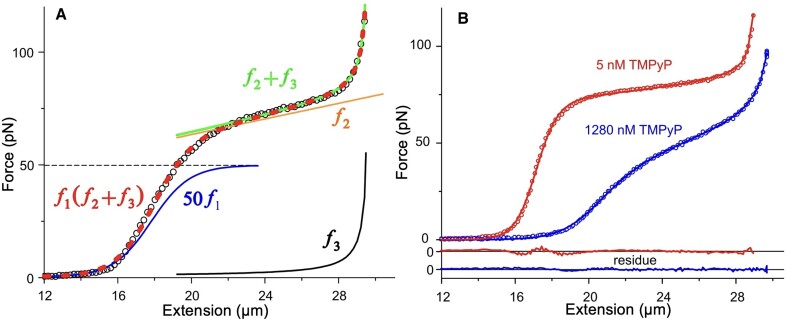
(**A**) Schematics of applying the empirical mathematical model to the force *versus* extension curves. The measured FEC is marked with open black circles. The model comprises a sigmoid (blue curve; 50*f*_1_ magnified for better visualization), a linear function (yellow curve; *f*_2_), and a hyperbolic (black curve, *f*_3_), so that the fitting function is *f*_1_(*f*_2_ + *f*_3_) (red dashed curve). *f*_2_ + *f*_3_ is shown with green continuous line. The equations of the functions are explained in the Materials and Methods. (**B**) Illustration of the goodness of fit in two extreme cases of TMPyP concentration, 5nM (red) and 1280 nM (blue). Extension was carried out at a NaCl concentration and pulling rate of 0.1 M and 20 μm/s, respectively. Open circles are the experimental data points, and the fits are marked with continuous lines. The colors of the residual traces correspond to those of the experimental data.

To dissect the effects of TMPyP, NaCl and pulling speed in detail, and to assign the effects to the structural and nanomechanical features of DNA, we developed an empirical mathematical model (Equations 1–4) to fit the FECs with (Figure 3). The fitting function (Equation 4) comprises three equations: a sigmoidal function (Equation 1) that describes the transition from the entropic regime to the overstretch transition *via* the enthalpic regime; a linear function (Equation 2) that describes the overstretch plateau itself; and a hyperbolic function (Equation 3) that describes the asymptotic behavior of the FEC approaching maximal extension (Figure 3A). The parameters (*P*_1-6_) of the equations scale with physical variables of DNA, as described in the Materials and Methods. Figure 3B shows the fit of the model to the FECs measured at extreme ends of the TMPyP concentration range (5 and 1280 nM). The results show that the empirical model developed here fits the experimental data remarkably well; therefore, plotting the fitting parameters as a function of TMPyP and NaCl concentrations and pulling rate is expected to reveal the mechanistic details of DNA’s nanomechanical response.

### Effect of TMPyP in the entropic regime

**Figure 4.**
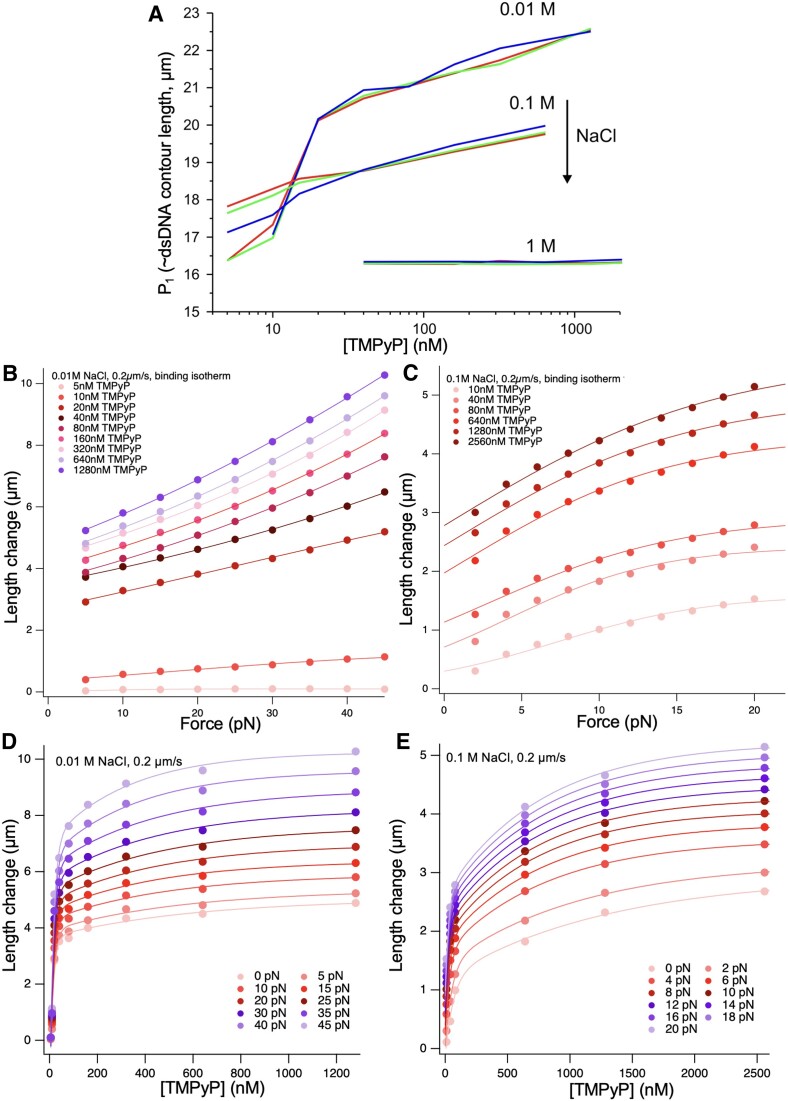
Effect of TMPyP on dsDNA length change in the entropic regime. (**A**) P_1_ (sigmoid step position which scales with dsDNA contour length) as a function of TMPyP concentration, pulling rate and NaCl concentration (0.01, 0.1 and 1M). The pulling rates of 0.2, 2, 20 μm/s are indicated with blue, green and red, respectively. (**B**, **C**) Length change of dsDNA as a function of force at different TMPyP concentrations, in the presence of 0.01 M and 0.1 M NaCl, at a pulling rate of 0.2 μm/s. The length change was calculated by subtracting the control (0 TMPyP) DNA length from the TMPyP-treated length measured at the given force. Data were fitted with Equation (8). Analyses of the fitting parameters (*K*_0_, Δ*x_eq_*, *n*) are shown in Supplementary Figure S1. (**D**, **E**) Length increment of dsDNA as a function of TMPyP concentration at different forces, in the presence of 0.01 M and 0.1 M NaCl, at a pulling rate of 0.2 μm/s. The zero-force data were obtained from the y-axis intercepts of Figure 4B and C. Data were fitted with double-exponential functions. Analyses of the apparent rate constants of the fitting functions are shown in Supplementary Figure S2.

**Figure 5.**
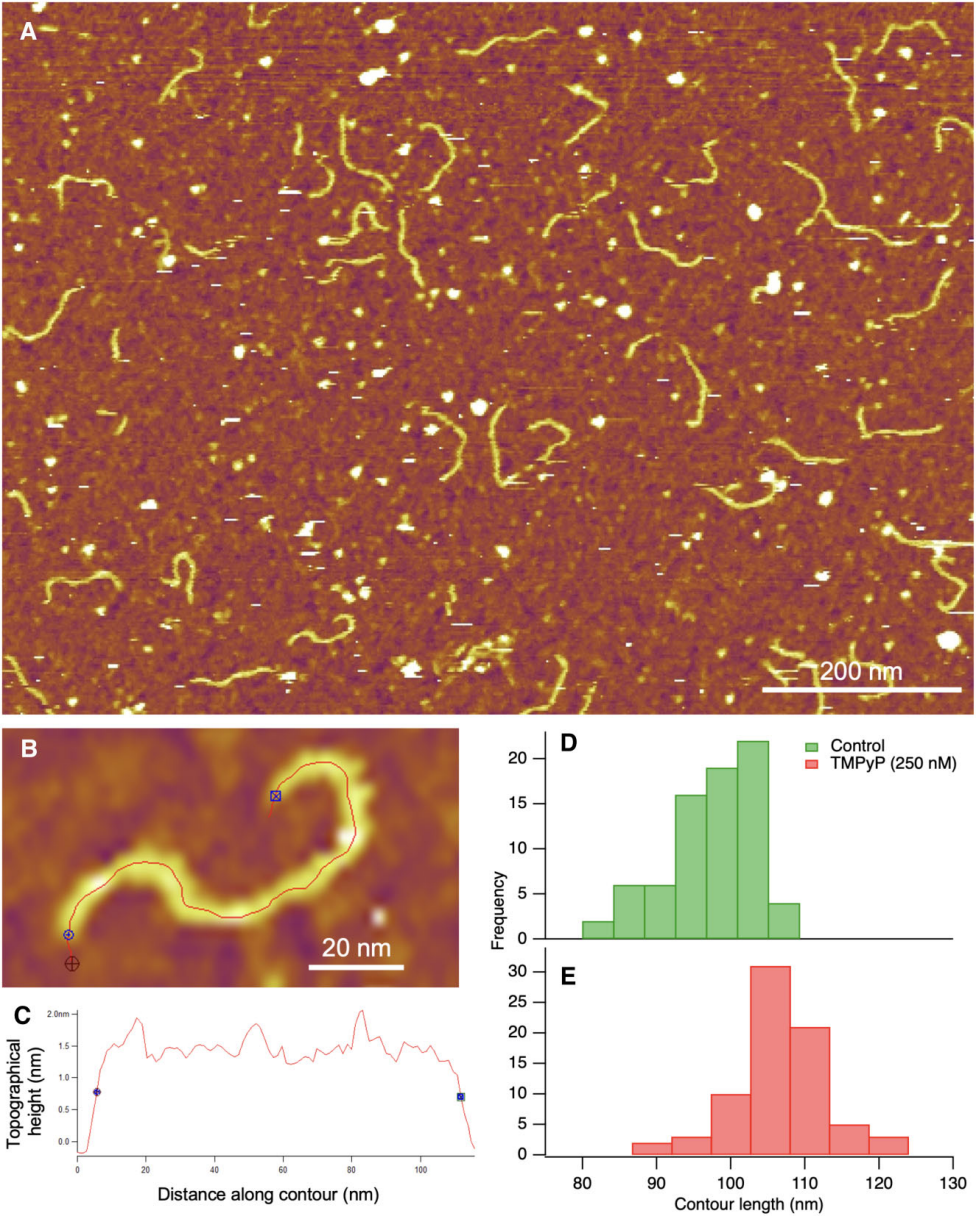
Effect of TMPyP on the contour length of conformationally relaxed dsDNA. (**A**) AFM image of 300-bp-long dsDNA molecules equilibrated to poly-l-lysine-coated mica surface and treated with 250 nM TMPyP. (**B**) Enlarged view of a single, surface-bound dsDNA molecule, with a trace drawn along its contour. (**C**) Topographical height *versus* axial distance curve measured for the dsDNA molecule in (B). The blue markers indicate the data points in between which the contour length of the molecule was identified. (**D**) Distribution of the contour length of control dsDNA molecules. (**E**) Distribution of the contour length of the TMPyP-treated (bottom) dsDNA molecules. The mean contour lengths of the control and TMPyP-treated DNA molecules were 97.31 nm (±6.01 nm S.D., *n* = 75) and 106.90 nm (±6.36 nm S.D., *n* = 75), respectively. The mean contour lengths are significantly different (mean difference 9.59 nm, Welch-test p-value 5.5 × 10^−17^). The mean end-to-end lengths of the control and TMPyP-treated dsDNA molecules were 68.84 nm (±18.89 nm) and 73.99 nm (±17.25 nm), respectively. From the relationship between the contur and end-to-end lengths (Equation 7) we calculated a persistence length of 10.8 and 12.8 nm for the control and TMPyP-treated dsDNA, respectively.

*P*_1_, which scales with the contour length of dsDNA, first increased rapidly, then slowly, as a function of increasing TMPyP concentration (Figure 4A). The effect was completely alleviated by increasing NaCl concentration to 1 M. Pulling rate had little effect on *P*_1_, detectable only at intermediate NaCl concentration (0.1 M) and at low (<40 nM) TMPyP concentrations. To investigate the TMPyP-induced DNA-lengthening effect in detail, we analyzed the length increment as a function of force (Figure 4B, C). The length increment increased monotonically as a function of force at every TMPyP concentration in the presence of both 0.01 M (Figure 4B) and 0.1 M NaCl (Figure 4C), although the trends of the data, indicated by fitting with a multi-site binding isotherm (Equation 8) (6), differed. Meaningful parameter values comparable with the binding of intercalating dyes (6) were obtained in the presence of 0.1 M NaCl only (Supplementary Figure S1). Altogether the results suggest that force enhances the binding of TMPyP to dsDNA. Extrapolating to zero force allowed us to calculate the TMPyP-induced DNA lengthening in the mechanically relaxed conformation, which helped comparing the results with independent structural measurements (see Figure 5) and further theoretical considerations (see Figure 7). dsDNA length increased double-exponentially as a function of TMPyP concentration in the presence of both 0.01 M (Figure 4D) and 0.1 M NaCl (Figure 4E), suggesting that TMPyP binds *via* two different mechanisms or steps with differing kinetic properties. In the low-force regime (0–20 pN) the characteristic TMPyP concentrations, at which a length change reaches 1/e times Δ*L*_max_ for both the fast and slow components of the double-exponential process, decreased with force, indicating that mechanical force sensitized dsDNA to further TMPyP binding (Supplementary Figure S2). Notably, the fast component is more than an order of magnitude faster than the slow one and occurs in the ten-nanomolar TMPyP concentration regime (Supplementary Figure S2).

We tested the DNA-lengthening effect of TMPyP by directly measuring the contour-length of surface-bound dsDNA molecules with AFM (Figure 5). We measured a 9.9% contour length increment in the presence of 250 nM TMPyP and 0.1 M NaCl, which is comparable to the 9.0% length increment calculated from the nanomechanical data extrapolated to zero force (Figure 4E) and taking the contour length of λ-DNA (16.37 μm) into account (see Figure 8B).

### Effect of TMPyP in the enthalpic regime

**Figure 6.**
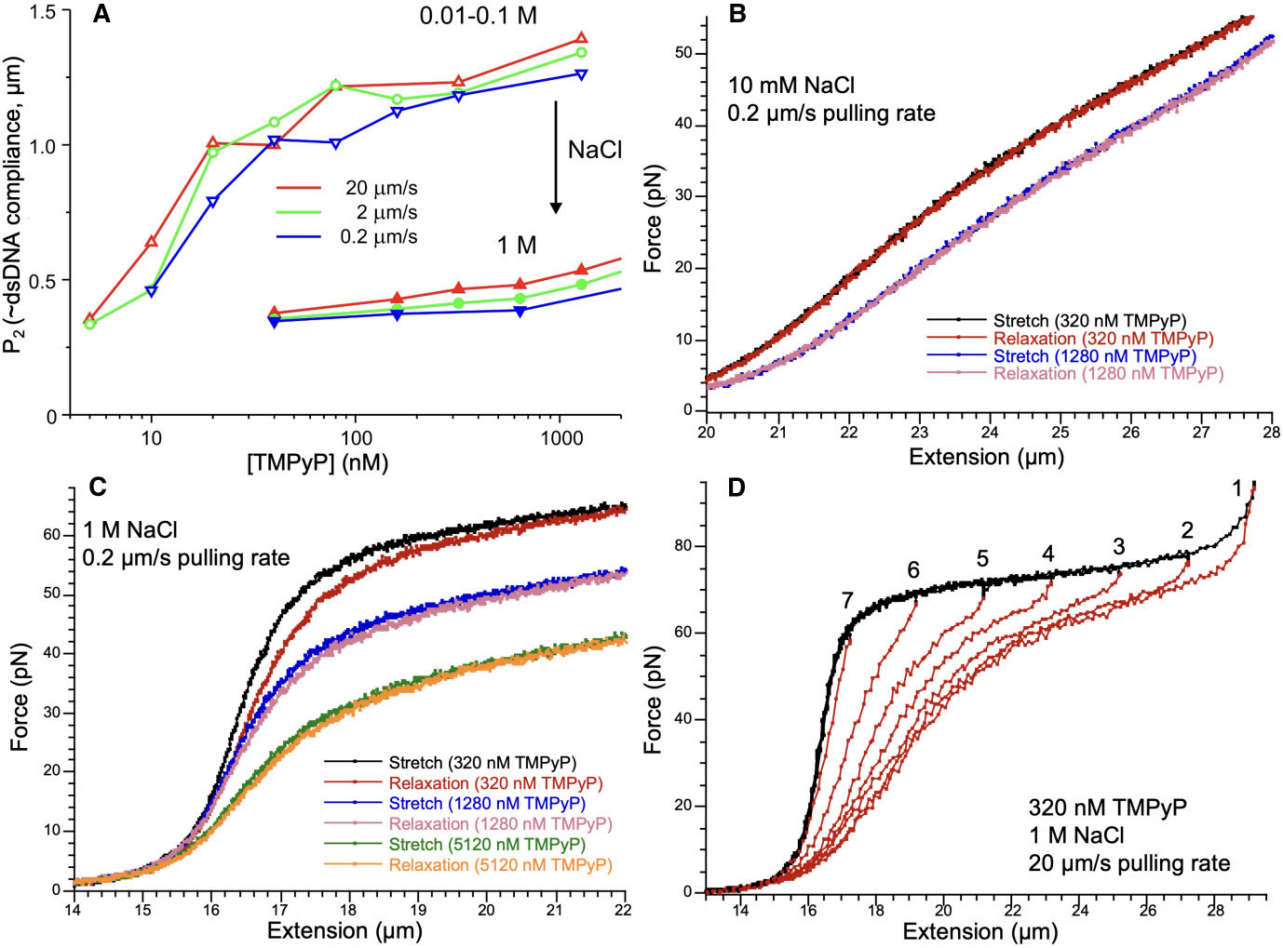
Effect of TMPyP on dsDNA nanomechanics in the enthalpic regime. (**A**) P_2_ (sigmoid step width, corresponding to dsDNA compliance) as a function of TMPyP concentration, pulling rate and NaCl concentration (0.01, 0.1 and 1M). The pulling rates of 0.2, 2, 20 μm/s are indicated with blue, green and red, respectively. 0.01–0.1 M indicates that the parameters in these two cases are close to each other within error. (**B**) Analysis of mechanical reversibility at 0.2 μm/s pulling rate in the presence of 10 mM NaCl and TMPyP concentrations indicated in the legend. The plot focuses on the enthalpic region. (**C**) Analysis of mechanical reversibility at 0.2 μm/s pulling rate in the presence of 1 M NaCl and TMPyP concentrations indicated in the legend. (**D**) Effect of maximal stretch on mechanical reversibility at 20 μm/s pulling rate in the presence of 1 M NaCl and 320 nM TMPyP. The DNA molecule was stretched and relaxed in consecutive mechanical cycles with progressively decreasing maximal stretch length. The points of maximum stretch length in the consecutice mechanical cycles are indicated with numbers above the force curves. The video of this experiment (Supplementary Video) is shown in the Supplementary Information.

*P*_2_, the width of the sigmoidal step, which scales with the apparent compliance of dsDNA (and inversely with its stiffness and stretch modulus), also increased rapidly, then slowly, as a function of increasing TMPyP concentration (Figure 6A). The effect was significantly reduced but not completely alleviated by increasing NaCl concentration to 1 M. Pulling rate had little effect on *P*_2_, detectable only at intermediate NaCl concentration (0.1 M) and at low (<40 nM) TMPyP concentrations. Considering that in the enthalpic regime the dsDNA structure becomes distorted, we tested whether the molecular system is in equilibrium by comparing the stretch and relaxation force curves (Figure 6B–D). At low NaCl concentration (0.01 M) and pulling rate (0.2 μm/s) we observed no force hysteresis across a wide TMPyP concentration range, indicating that the system was in thermodynamic equilibrium throughout the nanomechanical experiment (Figure 6B). Upon increasing NaCl concentration to 1 M, a small hysteresis appeared at low TMPyP concentration (Figure 6C). However, a systematic nanomechanical experiment, in which the maximum extension was progressively decreased, demonstrated that there is reversibility in the enthalpic regime, and hysteresis arises only if DNA has entered the overstretch transition (Figure 6D).

**Figure 7.**
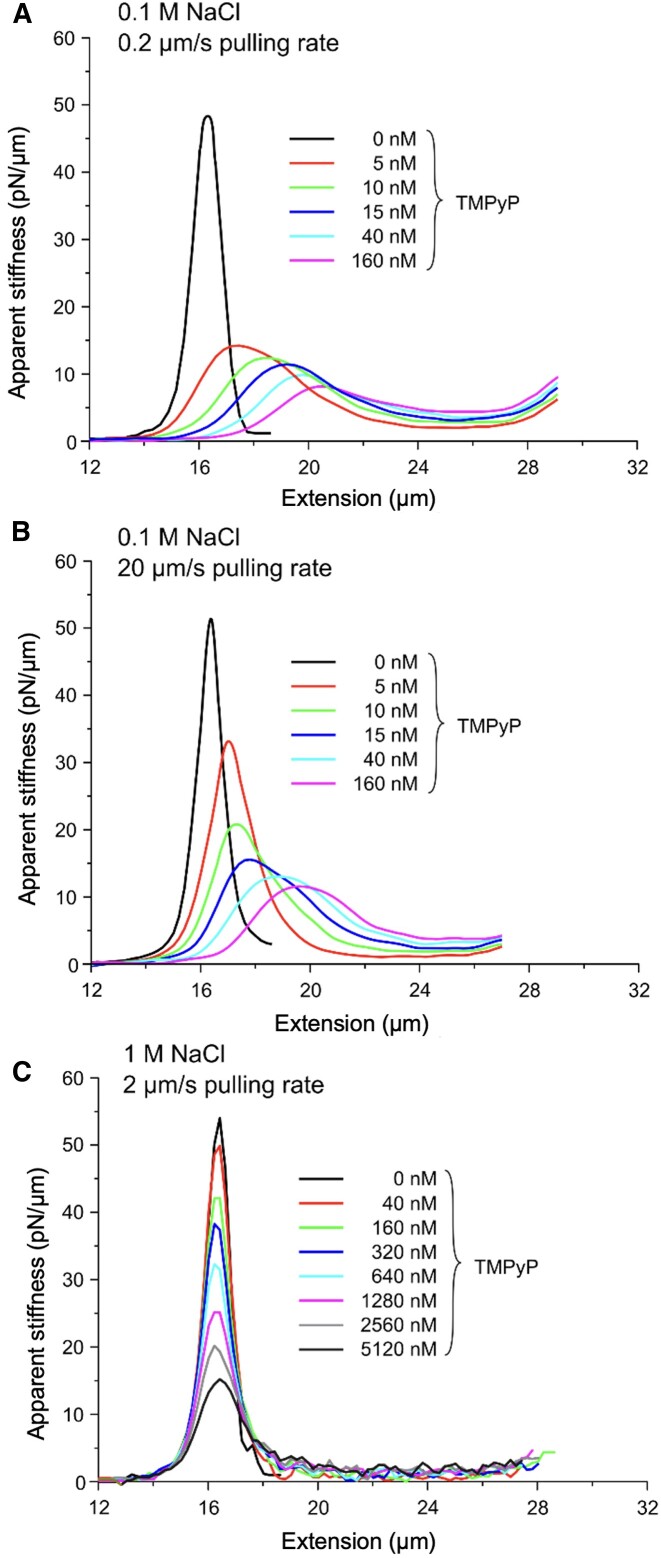
Instantaneous apparent stiffness of DNA generated by numerical derivation of stretch force–extension curves. Three series of TMPyP-dependent curves are shown: (**A**) at 0.1 M NaCl and 0.2 μm/s pulling speed, (**B**) at 0.1 M NaCl and 20 μm/s and (**C**) at 1 M NaCl and 2 μm/s. In the first two series (A and B) smoothing was applied, and in the third (C) the raw, unsmoothed data are shown. The colors correspond to the different TMPyP concentrations indicated in the legend.

While *P*_2_ reflects the apparent compliance of dsDNA, the apparent stiffness is a more meaningful and accessible measure of the instantaneous nanomechanical behavior. To calculate the apparent, instantaneous longitudinal stiffness of the DNA molecule and the effect of TMPyP on this characteristic, we numerically derivated the FECs (Figure 7). In the absence of TMPyP a narrow bell-shaped curve, centered at 16.2 μm, was observed, independently of the NaCl concentration or the pulling rate. The peak position, hence the inflection point of the sigmoidal function (Equation 1) coincides with the contour length of λ-phage DNA, substantiating the notion that the *P*_1_ parameter reflects the contour length of dsDNA in these experiments. The peak apparent stiffness of dsDNA is thus 50 pN/μm. Upon adding TMPyP at increasing concentrations, the curve broadened, the peak value decreased, and the peak position progressively shifted to increasing extensions (Figure 7A). At large pulling rates we observed a similar response, although the peak decrement and peak position shift were more gradual (Figure 7B). In the presence of 1 M NaCl the rightward shift of the peak was completely alleviated (Figure 7C), but increasing TMPyP concentrations continued to reduce peak stiffness.

**Figure 8.**
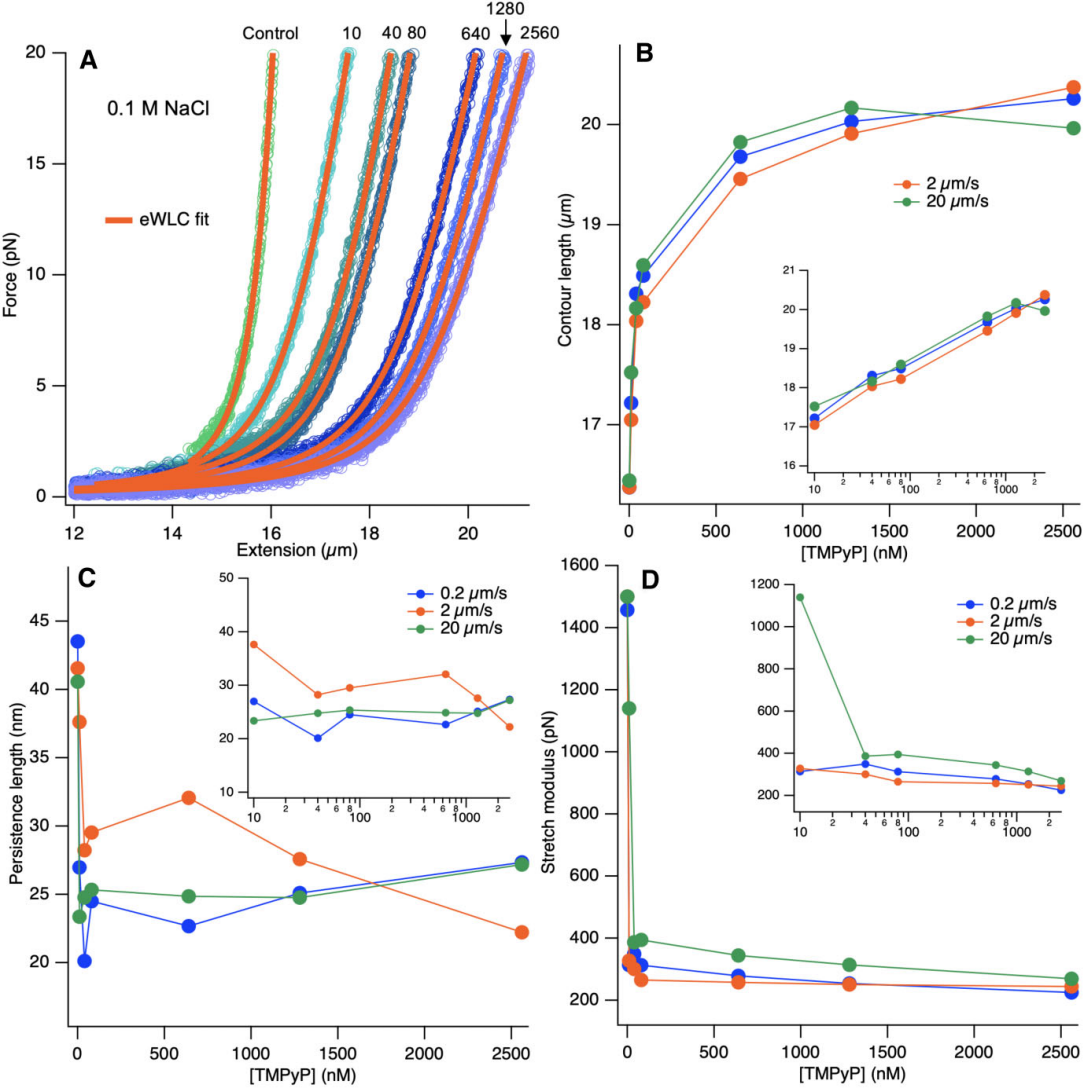
(**A**) Force versus extension curves of dsDNA fitted with the extensible wormlike-chain (eWLC) model (Equation 6) in the low-force regime (<20 pN). (**B–D**) Contour length, persistence length and stretch modulus, calculated from the eWLC model, as a function of TMPyP concentration. Insets of figures (B)–(D) show the respective data on log scale of TMPyP concentration.

To consider both the entropic and enthalpic contributions to dsDNA’s response to TMPyP binding, we carried out a systematic fitting of the extensible wormlike-chain (eWLC) model to the force *versus* extension data (Equation 6) (Figure 8). The contour length of dsDNA increased exponentially to a plateau of ∼20 μm as a function of increasing TMPyP concentration (Figure 8B). Persistence length and stretch modulus dropped rapidly at low TMPyP concentrations (<10 nM) and stabilized at around 25 nm and 300 pN, respectively (Figure 8C, D). Thus, dsDNA became more flexible and compliant upon TMPyP binding.

### Perturbation kinetics of TMPyP-DNA binding

**Figure 9.**
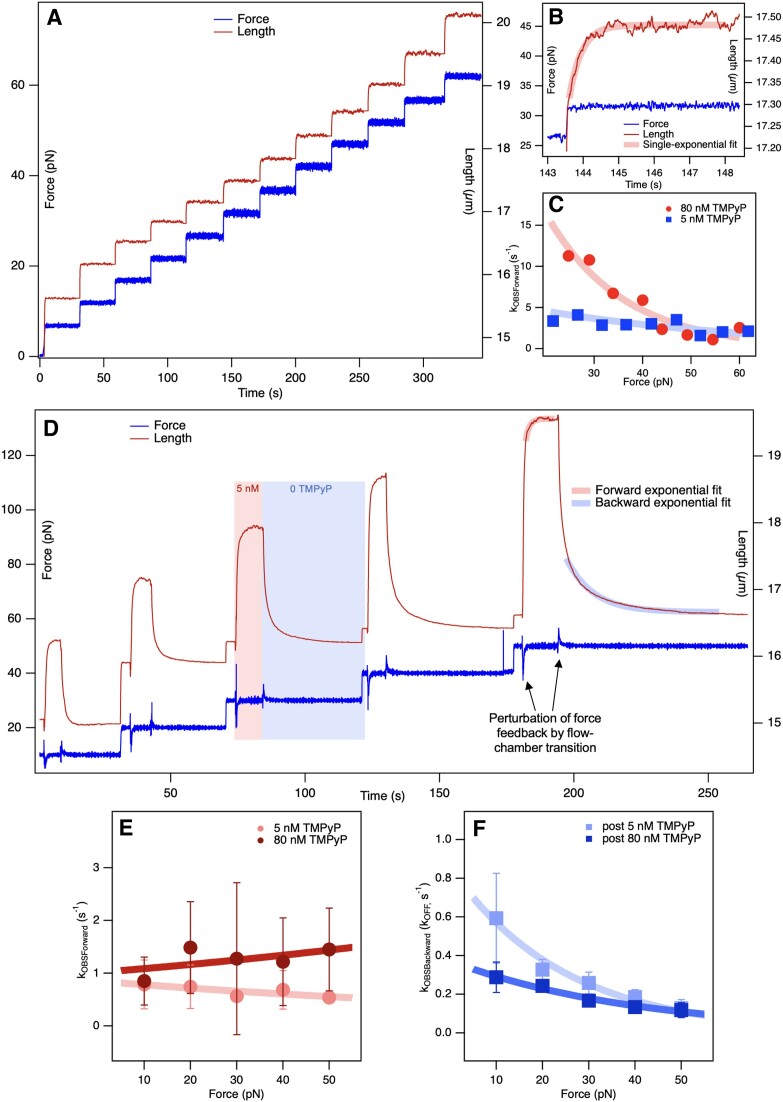
Perturbation kinetic analysis of dsDNA-TMPyP binding. (**A**) Force-jump experiment in the presence of 5 nM TMPyP. Force was increased in sudden 5-pN steps, then held constant with feedback. (**B**) dsDNA length data fitted with a mono-exponential function in the regime where force already stabilized at the setpoint. Notably, approximately 90% of the length increment has already occurred by this time point. (**C**) *k_OBS_* of the forward, TMPyP-binding reaction as a function of force, measured in the presence of 5 and 80 nM TMPyP. Data were fitted with Equation (9), from which we obtained *k*_0_ values of 7.1 (±0.7) and 57.9 (±17.5) s^−1^ for 5 and 80 nM TMPyP, respectively. Based on Equation (10), we calculated *k_ON_* and *k_OFF_* values of 0.68 nM^−1^s^−1^ and 3.7 s^−1^, respectively. (**D**) TMPyP concentration-jump experiment at constant forces set to 10, 20, 30, 40 and 50 pN. The dsDNA molecule, held between two beads at the given set force, was brought rapidly to 5 nM TMPyP, then back to 0 TMPyP (light red and blue shaded areas, respectively). The forward (high-TMPyP) and backward (0 TMPyP) length data, following the relaxation of the force after solution transfer, were fitted with mono-exponential functions (thick continuous light red and blue lines). (**E**) *k_OBS_* of the binding (forward) reaction as a function of force. *k_OBS_* values were calculated from the rate constants of the exponential fits to the high-TMPyP-concentration length data. The zero-force rates were 0.85 (±0.09) and 1.01 (±0.22) s^−1^ for 5 and 80 nM TMPyP, respectively. (**F**) *k_OBS_* of the dissociation (backward) reaction measured in 0 TMPyP, which corresponds to *k_OFF_*. *k_OFF_* values were calculated from exponential fits to the 0-TMPyP length data. The zero-force rates were 0.86 (±0.08) and 0.37 (±0.02) s^−1^ following exposure to 5 and 80 nM TMPyP, respectively. Error bars in (**E**)–(**F**) refer to standard deviation (SD) from four independent experiments.

To explore the mechanisms of TMPyP binding and dissociation to and from dsDNA in further detail, we carried out perturbation kinetic experiments on individual DNA molecules (Figure 9). Two types of experiments were systematically performed: (a) force-jump (Figure 9A–C) and (b) TMPyP-concentration jump (Figure 9D–F). In force-jump experiments the TMPyP-DNA binding equilibrium, established at either 5 or 80 nM TMPyP, was perturbed by rapidly stretching the DNA molecule and exposing it to progressively increasing forces incremented in 5-pN steps (Figure 9A). The force increment increased the DNA length, which eventually stabilized at a new equilibrium. Approximately 90% of the length increment took place during the force jump, but DNA length continued to increase past force stabilization and followed a mono-exponential function (Figure 9B). From the mono-exponential fits the force-dependent rates of length increase (*k_OBS_*) were calculated for both 5 and 80 nM TMPyP (Figure 9C). By fitting the data with Equation (9) we calculated *k*_0_ values of 7.1 (±0.7) and 57.9 (±17.5) s^−1^ for 5 and 80 nM TMPyP, respectively, which yielded *k_ON_* and *k_OFF_* values of 0.68 nM^−1^s^−1^ and 3.7 s^−1^, respectively (Equation 10).

In TMPyP concentration-jump experiments the DNA molecule, held at a pre-adjusted, constant force, was transferred rapidly from 0 TMPyP concentration to high TMPyP concentration (either 5 or 80 nM), and its length was allowed to relax, in force feedback, to a new value (Figure 9D, light-red-shaded area). Subsequently, the molecule was transferred rapidly back to 0 TMPyP, while continuing to maintain the constant force level and recording the length change as a function of time (Figure 9D, light-blue-shaded area). The time-dependent DNA-length changes following the relaxation of force perturbation could be well fitted with mono-exponential functions, from which the force-dependent rate constants of the TMPyP binding (*k*_OBSForward_) and dissociation (*k*_OBSBackward_) reactions could be obtained (Figure 9E and F). The zero-force rates of the binding reaction (*k*_0_), calculated from Equation (9) (Figure 9E) were 0.85 (±0.09) and 1.01 (±0.22) s^−1^ for 5 and 80 nM TMPyP, respectively. The zero-force rates calculated from the exponential fits to the TMPyP-dissociation length-change data (Figure 9F) were 0.86 (±0.08) and 0.37 (±0.02) s^−1^ for 0 TMPyP following exposure to 5 and 80 nM TMPyP, respectively.

### Effect of TMPyP on the overstretch transition

**Figure 10.**
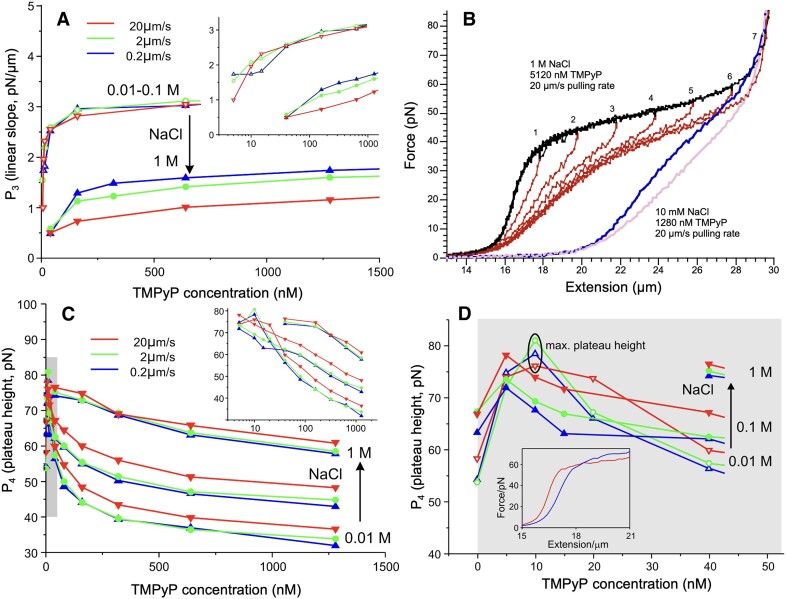
Effect of TMPyP on DNA nanomechanics in the overstretch plateau regime. (**A**) P_3_ (linear slope, scales inversely with overstretch cooperativity) as a function of TMPyP concentration, pulling rate and NaCl concentration (0.01, 0.1 and 1M). The pulling rates of 0.2, 2, 20 μm/s are indicated with blue, green and red, respectively. 0.01–0.1 M indicates that the parameters in these two cases are close to each other within error. Inset shows the data on log scale of TMPyP concentration. (**B**) Analysis of mechanical reversibility at 20 μm/s pulling rate in two different experimental conditions: 1 M NaCl and 5120 nM TMPyP, stretch and relaxation indicated in black and red, respectively; 10 mM NaCl and 1280 nM TMPyP, stretch and relaxation indicated in blue and pink, respectively. In the 1 M NaCl experiment the DNA molecule was stretched and relaxed in consecutive mechanical cycles with progressively increasing maximal stretch length. The points of maximum stretch length in the consecutive mechanical cycles are indicated with numbers above the force curves. (**C**) P_4_ (plateau height, corresponding to dsDNA stability), measured at an extension of 25 μm, as a function of TMPyP concentration, pulling rate and NaCl concentration (0.01, 0.1 and 1M). Inset shows the data on log scale of TMPyP concentration. (**D**) Expanded view of (C) in the TMPyP concentration range of 0–50 nM (see corresponding gray shaded areas). Plateau height peaks at a TMPyP concentration of 10 nM. **Inset**, force–extension curves at low TMPyP concentrations (10 mM NaCl, 0.2 μm/s pulling rate) shown to demonstrate the local peaking effect of TMPyP on the plateau height. Red and blue correspond to 5 and 10 nM TMPyP, respectively.

The overstretch transition is characterized by its slope and height, which are reflected in the *P*_3_ and *P*_4_ parameters of the fitting function, respectively. *P*_3_ increased rapidly, then slowly, as a function of TMPyP concentration (Figure 10A). The effect was significantly reduced, but it was not completely alleviated, upon increasing NaCl concentration to 1 M. At 1 M NaCl the pulling rate-dependence increased, suggesting that the molecular system shifted away from equilibrium. To test for this possibility, we compared the stretch and relaxation force curves at high (20 μm/s) pulling rates (Figure 10b). Indeed, a force hysteresis was present (black and red curves in Figure 10B), which progressively increased with increasing the extension across the overstretch transition. By contrast, at low NaCl (10 mM) the hysteresis was minimal (blue and pink curves).

*P*_4_, the height of the overstretch transition, measured systematically at the arbitrarily assigned extension of 25 μm, decreased progressively as function of TMPyP concentration (Figure 10C). Increasing NaCl concentration gradually reduced this effect. We also observed pulling rate-dependence, which concurs with its effect on the *P*_3_ parameter (see Figure 10B). Even though *P*_4_ decreased with increasing TMPyP concentration, below a TMPyP concentration of 10 nM we observed a transient increment and local maximum (Figure 10D). The observation is substantiated by the crossing of the FECs in the overstretch transition (Figure 10D, inset).

### Effect of TMPyP on the asymptotic regime

**Figure 11.**
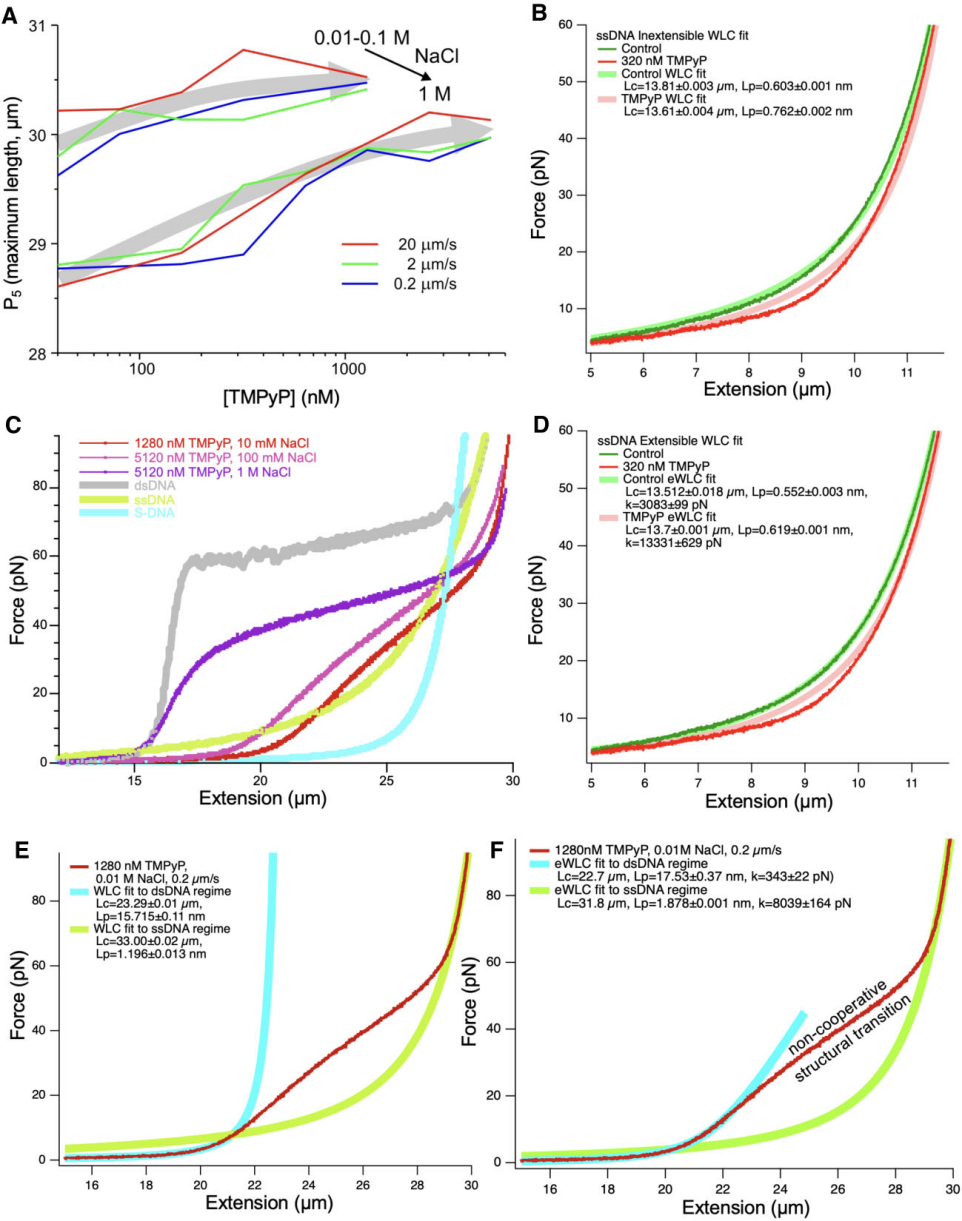
Effect of TMPyP on DNA nanomechanics in the asymptotic regime. (**A**) P_5_ (maximum molecular length) as a function of TMPyP concentration, pulling rate and NaCl concentration. 0.01–0.1 M means that the parameters in these two cases are close to each other within error. Gray arrows indicate the trend that P_5_ reaches a plateau. (**B**, **C**) FEC curves of ssDNA stretched in 0 and 320 nM TMPyP. Data were fitted with the inextensible wormlike-chain (WLC) and extensible wormlike-chain (eWLC) models. Fitting parameters are indicated in the legends. (**D**) Comparison of canonical, theoretical and extreme (maximum tested TMPyP concentrations) experimental force–extension curves of DNA. The experimental FECs are equilibrium traces obtained at low (0.2 μm/s) pulling rates. The dsDNA and ssDNA traces are experimental data, and the S-DNA is a simulated curve based on a modified extensible wormlike-chain model (95) The ssDNA FEC can be best fitted with an eWLC model (*L_C_*= 31.9 μm, *L_P_*= 1.1 nm, κ = 1642 pN). (**E, F**) Equilibrium FEC (red trace) of DNA obtained at 1280 nM TMPyP, 10 mM NaCl and 0.2 μm/s pulling rate with WLC and eWLC fits on the entropic (<7 pN, light blue) and asymptotic (>70 pN, light green) regimes. Fitting parameters are indicated in the legends.

*P*_5_, which reflects the maximal length of the overstretched DNA molecule, increased gradually towards a maximum as a function of TMPyP concentration (Figure 11a), suggesting that TMPyP may bind directly to ssDNA, leading to its extension. In 1 M NaCl, *P*_5_ was reduced considerably at low TMPyP concentration, then it increased apparently towards the same maximum value. Pulling rate had minimal effect on this parameter and on its NaCl concentration dependence. To test whether TMPyP indeed binds to ssDNA, we measured the nanomechanical behavior of single ssDNA molecules stretched and relaxed in the presence of 320 nM TMPyP (Figure 11B, C). In the 0–60 pN force range the TMPyP-treated ssDNA molecules were more extended, and their FEC could be better fitted with the eWLC model. The model fitting indicated that TMPyP treatment increased the contour length, the persistence length and the stretch modulus of ssDNA (Figure 11c). Notably, between 10 and 20 pN, the FEC of the TMPyP-treated ssDNA deviated from the model curves, raising the possibility that a mechanically-driven structural change occurs in this regime.

Finally, we probed the nature of the TMPyP-saturated DNA molecule by comparing its FEC with theoretical models (WLC and eWLC) (Figure 11D–F). We used the FEC of DNA streched in the presence of 1280 nM TMPyP and 0.01 M NaCl, as this was farthest away from the control nanomechanical curve (Figure 11D). While the TMPyP-saturated DNA is in an over-extended form, its mechanical behavior differs from that of the corresponding ssDNA and S-DNA (Figure 11D). In the low-force regime (<20 pN) it is best described as a compliant, extensible WLC with a contour length pre-extended by up to 39% (Figure 11F). In the high-force regime (>60 pN) it is an eWLC with a contour length identical to that of the corresponding ssDNA of the overstretched λ-phage DNA (*L_C_* = 31.9 μm) but with increased persistence length and stretch modulus.

## Discussion

### TMPyP binds to dsDNA by two modes and elongates it

In the present work we investigated the effect of TMPyP, a chemical widely used in photodynamic therapy (25,27,33,57–62) and G-quadruple stabilization (41,63–69), on DNA nanomechanics. Even though the interaction of TMPyP with DNA has been investigated extensively, how it might alter the nanomechanical behavior of DNA has remained unknown. Because many important DNA-binding proteins are mechanoenzymes (e.g. DNA- and RNA-polymerases, etc.), understanding DNA’s nanomechanical response to pharmacological perturbations is of great importance.

The addition of TMPyP to dsDNA in increasing concentration resulted in a complex array of changes (Figure 2) with respect to DNA’s canonical force versus extension curve (FEC) (Figure 1). Moreover, adding NaCl to the molecular system, which is commonly used for stabilizing the double-helical structure and for electrostatic screening (4,6), resulted in further, complex changes in the FEC. Because in nanomechanical experiments force is used to distort molecular structure as a function of time, and as a result the equilibrium of the binding reaction may be constantly shifted, the thermodynamic state of the system is an important question. On one hand, it may be desired to characterize DNA in a chemically constant system, in which the ratio of the reactant to DNA in the complex remains steady (6). We found that the binding of TMPyP to DNA is very fast on realistic time scales of nanomechanical experiments (see perturbation kinetics experiments), which precluded the characterization of a molecular system in which the ratio of DNA-bound TMPyP molecules remained constant. On the other hand, it may be desired to characterize DNA in thermodynamic equilibrium, in which the TMPyP–DNA complex is in chemical and conformational equilibrium throughout the mechanical stretch-relaxation cycle. Such an equilibrium is characterized by the absence of force hysteresis (70). Increasing the pulling rate pushes the molecular system away from equilibrium, and *vice versa*. To test for equilibrium, we therefore exposed the TMPyP-DNA complex to different pulling rates, which resulted in a large, multi-dimensional dataset (Figure 2).

To sort between the types of effects TMPyP and NaCl may have on DNA, we introduced an empirical mathematical model with which we fitted the FECs (Figure 3). The significance of this simple model lies in the fact that its parameters reflect the physical characteristics of DNA. Accordingly, we were able to follow changes in the contour length and compliance of dsDNA, the average force and cooperativity of the overstretch transition, and the maximal length of overstretched DNA.

TMPyP caused a significant, up to 37% increase in the contour length of dsDNA, reflected in the change of the *P*_1_ parameter (Figure 4A), in an essentially pulling rate-independent manner, indicating that the structural changes caused by TMPyP take place rapidly. Most of the lengthening occurs in the low TMPyP concentration range (<40 nM), followed by a more gradual TMPyP concentration-dependent extension, suggesting that TMPyP binds in at least two binding modes (43,45) with different TMPyP concentration sensitivities and possibly kinetics. The length increment could be completely inhibited by 1 M NaCl, indicating that the mechanism of TMPyP binding to DNA is electrostatic. It has been shown before that TMPyP intercalates into DNA (71–74), and that intercalators cause DNA lengthening (6,8,10,12,13,53–56). Thus, we conclude that the length increment is caused primarily by TMPyP intercalation. It has been also found that TMPyP binds to DNA with alternative mechanisms, major and minor groove binding (72,75,76), likely in a highly dynamic equilibrium. Most plausibly, intercalation and the additional binding mechanisms altogether lead to DNA lengthening. To dissect the binding mechanisms further, we measured the length increment at given forces in an analysis employed before in the investigation of intercalator-DNA interactions (6). The force-dependent length change could be fitted with a multi-site binding isotherm (Equation 8) (6) in the presence of low (0.01 M) and high (0.1 M) concentration of NaCl (Figure 4b-c). Thus, we could estimate equilibrium binding parameters in spite of the fact that, in contrast to cyanine intercalators, the amount of DNA-bound TMPyP was not possible to measure with fluorescence microscopic methods due to its very low quantum yield that is even reduced upon DNA binding (28). Meaningful fitting parameters were obtained in the case of 0.1 M NaCl (Supplementary Figure S1), which are comparable with data obtained earlier for cyanine-dye intercalators (6). The average affinity constant (*K*_0_) and the equilibrium length change caused by the binding of a single TMPyP molecule (Δ*x_eq_*) were 4.6 × 10^5^ M^−1^ and 0.64 nm, respectively. The apparent TMPyP footprint decreased with increasing concentration, showing striking similarity to earlier spectroscopic results (Supplementary Figure S1f inset) (77). Even in 0.1 M NaCl, however, *K*_0_ and Δ*x_eq_* displayed a surprising TMPyP concentration dependence (Supplementary Figure S1d-e). Altogether, the binding-isotherm fits suggest a complex TMPyP–DNA binding mechanism involving, possibly, multiple binding modes, reaction intermediates and the role of TMPyP-induced DNA structural change (e.g. premature beginning of the overstretch transition). Extrapolation to zero force allowed us to calculate the length change of dsDNA induced by TMPyP binding in mechanically relaxed conditions, which supports prior observations obtained in ensemble measurements (37,38,61). The TMPyP concentration-dependent DNA length change data could be fitted with double-exponential functions in the case of both low (0.01 M) and high (0.1 M) NaCl concentrations (Figure 4d-e), supporting the notion that TMPyP binding occurs via complex mechanisms. In the low-force regime the characteristic TMPyP concentration, at which a length change of 1/e×Δ*L*_max_ occurs, decreased with force (Supplementary Figure S2). Altogether, mechanical force makes room along DNA for additional TMPyP, but it also sensitizes DNA for further TMPyP binding.

The DNA-lengthening effect of TMPyP could be well substantiated with AFM measurements (Figure 5). Although the 9.9% contour-length increment found in the AFM experiments is similar to the 9.0% length increment calculated from nanomechanical measurements under similar buffer conditions, we note that the latter is not identical to the contour-length change which can be calculated from theoretical model fits and was 14.4% for the given experimental conditions (Figure 8). The smaller value of the contour-length increment calculated in the AFM experiments versus the theoretical model fits (9.9% versus 14.4%) is due most likely to constraints imposed by the binding of DNA to the substrate surface. Accordingly, the calculated persistence lengths (50) for the control and TMPyP-treated dsDNA were 10.8 and 12.8 nm, respectively, which indeed suggests that DNA was kinetically trapped on the substrate surface. Altogether, however, the AFM measurements provided an independent structural proof for the dsDNA-lengthening effect of TMPyP.

### TMPyP binding increases apparent dsDNA compliance

TMPyP caused a large, nearly step-like increase in dsDNA compliance within a relatively narrow concentration range (10–40 nM), as judged from the change in the *P*_2_ parameter (Figure 6a). The slight pulling rate-dependence of the *P*_2_ parameter suggested that the molecular system may not be in equilibrium. The DNA-softening effect of TMPyP could be almost completely reversed by raising the NaCl concentration to 1 M. Upon stretching DNA with force further and further the TMPyP molecules keep binding (see Figure 4b-c), hence the system is in progressive chemical change. Therefore, the molecular system is not a true elastic body, and viscous behavior may arise depending on the kinetic and thermodynamic state of the TMPyP- and Na-DNA binding reactions. We tested for thermodynamic equilibrium, and found that it prevails as long as DNA is prevented from entering the overstretch transition (Figure 6D). Interestingly, NaCl has a differential effect on dsDNA in this region of the FEC: whereas the original contour length of dsDNA is completely recovered, the softening effect of TMPyP persists even at high (1 M) NaCl concentration (Figure 7C), suggesting that NaCl competes differently with TMPyP binding, depending on the binding mechanism. Altogether, dsDNA behaves as an apparent elastic body, the stiffness of which can be sensitively modulated by TMPyP (in the nM concentration range) and by ionic strength. Fitting the extensible wormlike-chain (eWLC) model to the experimental data (Figure 8) successfully re-capitulated the effects of TMPyP in both the entropic and enthalpic regions, pointing further at the complex DNA-lengthening effect (Figure 8B) and the sensitive DNA-softening effect in the nanomolar TMPyP regime (Figure 8D).

### Perturbation kinetics reveal a dynamic TMPyP-DNA binding equilibrium

By employing perturbation kinetic experiments on single DNA molecules we were able to uncover the dynamics of TMPyP-DNA interaction. In force-jump experiments (Figure 9A–C) the chemical equilibrium is pushed slightly out of equilibrium by mechanical force, and the re-establishment of the new equilibrium is governed simultaneously by both the forward (binding) and reverse (dissociation) processes. By contrast, in concentration-jump experiments (Figure 9D–F) the molecular system in mechanical equilibrium is rapidly positioned into drastically different chemical environments (high and zero concentration TMPyP), allowing us to push the system so far away from equilibrium that only one of the processes dominates, at least initially. We note here that when the TMPyP–DNA system is brought into 0 TMPyP, only dissociation occurs throughout the observation time window, because constant-velocity buffer flow washes away every unbound TMPyP molecule (see Materials and Methods), thereby preventing any accumulation of free ligand and hence the onset of new binding. Thus, while the concentration-jump single-molecule experiment at 0 TMPyP permits the direct estimation of *k_OFF_*, both binding and dissociation contribute to the observed kinetics when approaching equilibrium at high TMPyP concentration (5 or 80 nM). The two different perturbation kinetic approaches gave somewhat different results, suggesting that equilibrium may be reached *via* different pathways in these different experimental conditions. The *k_ON_* and *k_OFF_* values calculated from force-jump experiments (Figure 9C, Equation 10) were 0.68 nM^−1^s^−1^ and 3.7 s^−1^, respectively. By contrast, the *k_ON_* and *k_OFF_* values calculated from concentration-jump experiments from zero to high TMPyP were 0.002 nM^−1^s^−1^ and 0.84 s^−1^, respectively (Figure 9E, Equation 10). The *k_OFF_* values calculated directly from exponential fits to concentration-jump experiments from either 5 or 80 nM TMPyP to 0 TMPyP were 0.86 and 0.37 s^−1^, respectively (Figure 9F), which are comparable to the value obtained from the forward concentration-jump measurements (Figure 9E). The TMPyP concentration dependence of *k_ON_* and *k_OFF_* seen in the experiments require further investigation, and may be related to the complexity of the binding reaction and structural changes evoked in DNA. Notably, the force-dependent increment in the amount of TMPyP bound to DNA in equilibrium (see Figure 4B, C) may be due to the force-dependent reduction of *k*_OFF_ (Figure 9F).

### TMPyP binding reduces the cooperativity and overall force of overstretch transition

Upon reaching a threshold force, typically ∼60 pN, under conditions resembling physiological, dsDNA goes through a cooperative overstretch transition characterized by a significant (>60%) lengthening that occurs within a narrow (∼15 pN) force range (4). Three main processes are thought to occur during this transition, the ratios of which are influenced by the number of nicks along DNA and environmental parameters such as ionic strength (78,79): conversion of B-DNA to S-DNA, melting bubble formation and strand unpeeling. Because these processes are affected by the strength of association between the DNA strands, the average plateau height is thought to reflect the stability of the double-stranded DNA structure. The cooperativity of the transition is manifested in the narrowness of the force range, hence the inverse of the FEC slope in this regime, and is related to the processes running linearly along the contour of the DNA chain. We found that the overstretch transition was greatly altered by the addition of TMPyP and then NaCl (Figure 10). Cooperativity, reflected in the inverse of the *P*_3_ parameter, was significantly reduced within a narrow TMPyP range (0–40 nM), then continued to decrease as a function of increasing TMPyP concentration (Figure 10a). Conceivably, the TMPyP molecules that bound to dsDNA remain attached throughout the transition and act as road-blocks that inhibit the progression of the molecular changes along the chain. Increasing NaCl concentration to 1 M partially restored cooperativity, and a strong pulling-rate dependence was present. Considering that 1 M NaCl restores the contour length of dsDNA completely (Figure 4A), in the presence of high TMPyP and NaCl concentrations the roadblock TMPyP molecules are likely ones that bind newly to DNA during the overstretch transition, plausibly to ssDNA regions. NaCl competes inefficiently with these newly bound roadblock TMPyP molecules which also inhibit the re-formation of dsDNA, resulting in a marked force hysteresis (Figure 10B, black and red traces). At high TMPyP but low NaCl concentrations (Figure 10B, blue and pink traces) a lengthened dsDNA and very little hysteresis are observed, which raises the possibility that the intercalated/groove-bound TMPyP population can be converted directly into the ssDNA-bound one.

The average force of the overstretch transition, reflected in the *P*_4_ parameter, decreased progressively as a function of increasing TMPyP concentration, and the effect was partially restored by increasing the concentration of NaCl (Figure 10C). Interestingly, however, the *P*_4_ decrease was not monotonic, but a local maximum was observed at 10 nM TMPyP (Figure 10D). This surprising finding suggests that the local chemical equilibria of the different binding modes become re-arranged in between the reactions stabilizing and de-stabilizing dsDNA. Conceivably, in the low TMPyP concentration range the stabilizing effects of intercalation and groove binding dominate (37,38,43), whereas at higher TMPyP concentrations the de-stabilizing effects of ssDNA binding become overwhelming. In support, the interaction with ssDNA-binding proteins results in similar FECs (13,22,23). Furthermore, it has been shown that the binding of actinomycin D (ActD) to DNA, which may occur *via* intercalation between dsDNA base pairs (80–84), ssDNA association (85–88) and ssDNA base intercalation (89,90), results in the lowering of the average overstretch force and reduction of cooperativity (91). Altogether, the complex array of TMPyP- and NaCl-induced effects on the overstretch transition of DNA is determined by a shift between the stabilizing and de-stabilizing TMPyP-DNA binding modes, and by the differential screening of intercalating and non-intercalating TMPyP-DNA association by NaCl.

### TMPyP binds to ssDNA and elongates it

Upon reaching extreme stretch, dsDNA is eventually converted into ssDNA, in which the strands are held together by a small number of hydrogen bonds. Thus, in the asymptotic regime (Figure 1) the FEC is set by the properties (contour and persistence lengths) of ssDNA. Our observations on the effects of TMPyP on the overstretch transition already raised the possibility that TMPyP is able to bind ssDNA directly (Figure 10). Upon the addition of TMPyP in increasing concentrations, the contour length of ssDNA, reflected in the *P*_5_ parameter, increased gradually towards a maximum (Figure 11A). At a NaCl concentration of 1 M, the contour length increment towards the same maximum was more pronounced. In other words, at high concentrations of NaCl ssDNA is contracted, and TMPyP competes with NaCl, leading to the lengthening of the molecule. The NaCl-induced contraction is likely caused by a decrease in the electrostatic persistence length of ssDNA due to electrostatic screening by Na^+^ ions. The competing effect by TMPyP is then plausibly caused by the intercalation of the positively charged molecules in between the bases of ssDNA. As estimated from the FECs, the binding of TMPyP at high concentrations results in the lengthening of ssDNA by >3%. Independent nanomechanical experiments on ssDNA molecules clearly demonstrated that TMPyP can interact directly with ssDNA (Figure 11B, C).

### Possible structure of the TMPyP-bound DNA molecule

TMPyP binding in different modes combined with mechanical force converts DNA into a yet unknown structure. To estimate the structure, we compared the FEC of DNA in high TMPyP concentration with the extreme scenarios of the control dsDNA, ssDNA and S-DNA (Figure 11D). At low (10–100 mM) NaCl and high (>1280 nM) TMPyP concentrations a highly extended and compliant dsDNA is seen, which is converted by a non-cooperative force-driven transition into a structure that appears longer, in the 60–90 pN force range, than the control ssDNA. 1 M NaCl restores the contour length and some of the stiffness of dsDNA, which is converted by a more-or-less cooperative transition into a similarly overstretched structure. We exclude the possibility that any part of the length change might be due to G-quadruplexes, which are known to be stabilized by TMPyP (40,41,67,92). Even though λ-phage DNA is predicted to contain 30 G-quadruplex sequences (Supplementary Figure S3), their formation would require *a priori* dsDNA denaturation. Furthermore, the mechanically-driven rupture of G-quadruplexes is expected to result in force sawteeth at low forces (93,94), which we have not observed in the FECs. To investigate the nature of the TMPyP-bound DNA further, we fitted the low- (<7 pN) and high-force (>70 pN) sections of the FEC with the inextensible wormlike-chain (WLC) model (Equation 5) (47) (Figure 11E) and the extensible wormlike-chain (eWLC) model (Equation 6) (48,49) (Figure 11f). At low forces the TMPyP-bound DNA behaves as a compliant eWLC that, based simply on the reduced persistence length (*L_P_* between 15.7 and 17.5 nm) resembles an S-DNA (95). At high forces it behaves as a ssDNA (*L_P_* between 1.2 and 1.9 nm) with a contour length that is in fact identical to that of one strand of λ-phage DNA. The departure of the FEC of the TMPyP-treated ssDNA from that of the mechanically-induced free ssDNA (see Figures 1 and 11D) is due to its longer persistence length, which is caused by the TMPyP molecules remaining attached to ssDNA in spite of the mechanical load. The mechanically-driven non-cooperative structural transition then converts the molecule reversibly, hence without TMPyP dissociation, from one state to the other.

Finally, it is worth pointing out that the employed TMPyP concentrations fall below or well within those used in *in vitro* photodynamic therapy (57,60,96). Therefore, the structural and nanomechanical changes documented here are highly relevant during the therapeutic applications of TMPyP. Furthermore, the largest amplitude of the nanomechanical changes in DNA are evoked in a relatively narrow nanomolar (5–40 nM) TMPyP concentration range. Thus, an interplay between nanomolar TMPyP concentrations and piconewton forces may tune DNA’s structural and nanomechanical characteristics, thereby controlling the wide array of DNA-associated mechanoenzymatic processes.

## Conclusions

Here, we have uncovered a complex array of TMPyP-induced nanomechanical changes in DNA. TMPyP binds to dsDNA in a highly dynamic process and leads to its significant lengthening. Force increases the amount of TMPyP bound in equilibrium but decreases the kinetic rate constants of the binding and dissociation reactions. TMPyP binding reduces the apparent instantaneous stiffness of dsDNA. TMPyP initially (at low concentrations) stabilizes, then (at high concentrations) destabilizes dsDNA. The cooperativity of the overstretch transition is reduced due to road-blocks that slow the transition. TMPyP binds to ssDNA, thereby lengthening it. ssDNA-bound TMPyP inhibits the re-formation of dsDNA. NaCl efficiently competes with TMPyP for DNA binding, but differentiates between the lengthening and stabilizing processes. As a result, the dsDNA contour length is efficiently recovered, but the rest of the TMPyP effects partially remain. At high NaCl concentrations, ssDNA contour length is reduced, most likely due to electrostatic screening, and TMPyP competes with NaCl by screening its length-reducing effect. The complex, TMPyP concentration-dependent changes in DNA nanomechanics provide a wide array of possibilities to modulate the force-dependent processes within the genome and may have significant therapeutic implications.

## Supplementary Material

gkae560_Supplemental_Files

## Data Availability

All experimental data are available upon request by writing to the corresponding author.
