# Supplementary material for: TMPyP binding evokes a complex, tunable nanomechanical response in DNA
Source: Nucleic Acids Res. 2024 Jun 29;52(14):8399–418. doi: 10.1093/nar/gkae560 (PMC11317170; doi:10.1093/nar/gkae560)
Supplement: gkae560_Supplemental_Files [file gkae560_supplemental_files.zip › TMPyP_MS_SI_20240601.pdf]

Supplementary Information

TMPyP binding evokes a complex, tunable nanomechanical response in DNA

Balázs Kretzer[1,2], Levente Herényi[1], Gabriella Csík[1], Eszter Supala[1], Ádám Orosz[1], Hedvig Tordai[1], Bálint Kiss[1,2] and Miklós Kellermayer[1,2,*]

[1]Department of Biophysics and Radiation Biology, Semmelweis University, Tűzoltó Str. 37-47, H1094 Budapest, Hungary
[2]HUNREN-SE Biophysical Virology Group, Tűzoltó Str. 37-47, H1094 Budapest, Hungary

* To whom correspondence may be addressed at:
kellermayer.miklos@ semmelweis.hu
Tel.: +36-20-825-9994

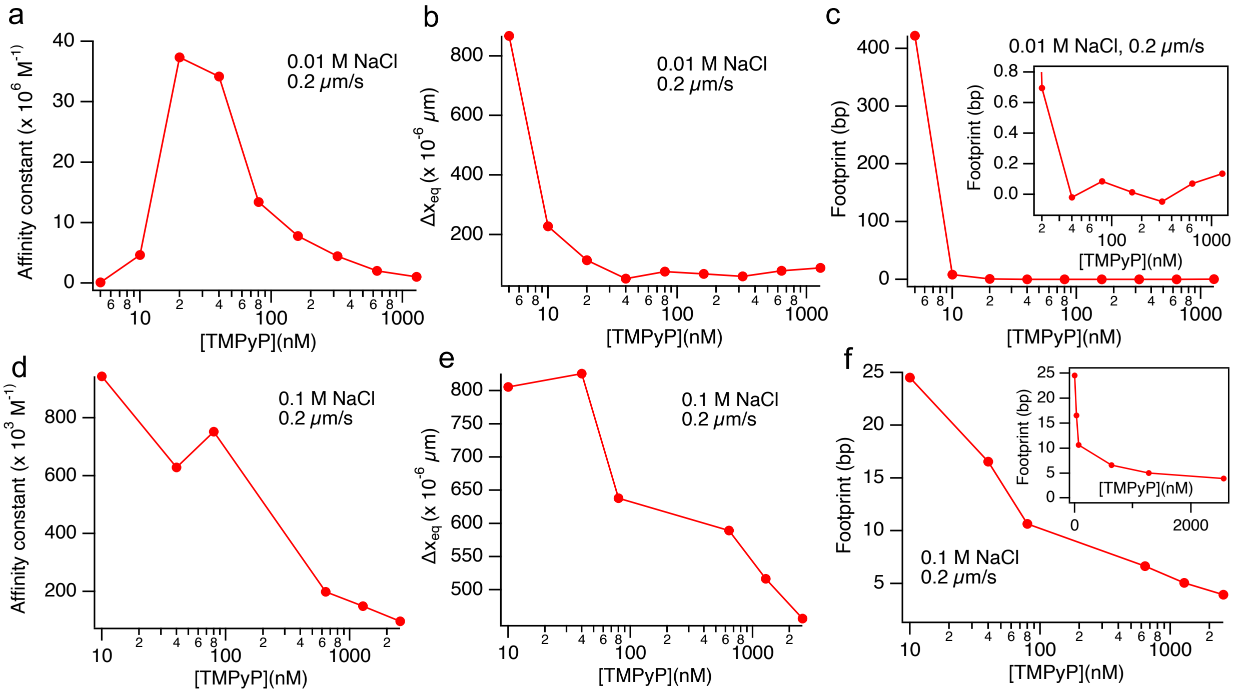

Figure S1. Parameters of the multi-site binding isotherm (eq8, Materials and Methods of the main text) as a function of TMPyP concentration. Affinity constant at zero force (K_0) **(a, d)**, the equilibrium length change caused by the binding of a single TMPyP molecule (Δx_{eq}) **(b, e)** and the apparent TMPyP footprint (n, number of base pairs along DNA per TMPyP molecule) **(c, f)** are shown for pulling rates of 0.2 μm/s and either 0.01 M (upper row) or 0.1 M NaCl (lower row). **Inset** of **c)** shows the footprint values in the TMPyP concentration range of 20-1280 nM. **Inset** of **f)** shows the footprint data on linear scale of TMPyP concentration.

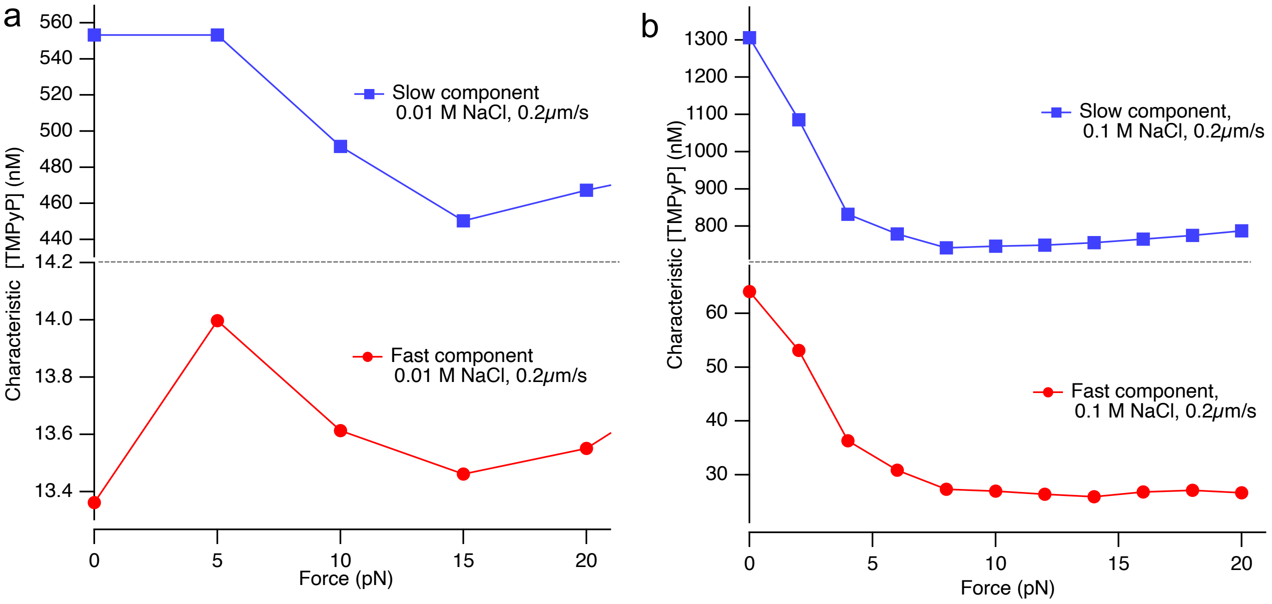

Figure S2. Characteristic TMPyP concentration as a function of force calculated from double-exponential fits to the length change *versus* TMPyP concentration data obtained in the presence of 0.01 M (**a**) and 0.1 M NaCl (**b**). The fast and slow components of the double-exponential fits are displayed separately. The characteristic TMPyP concentration is the one at which ($\Delta L = 1/e \times \Delta L_{max}$) change in the length of dsDNA occurred at the given force. Data are shown in the force range of 0-20 pN. The characteristic TMPyP concentrations were derived from the results shown in **Fig.4d and e** of the main text.

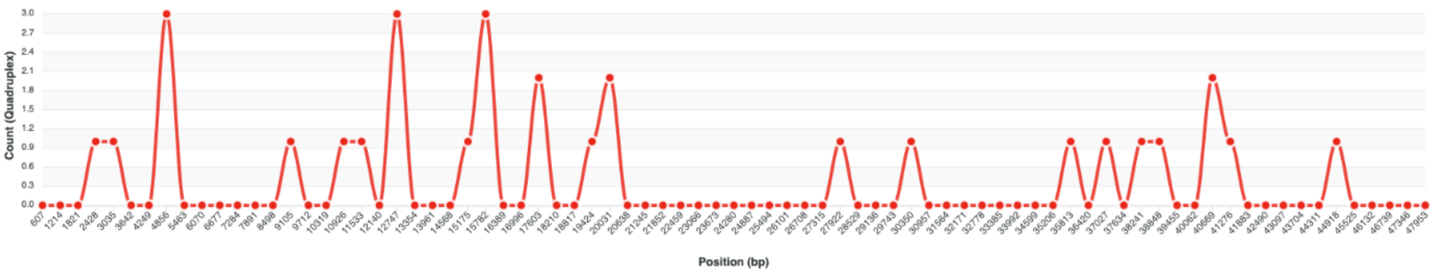

Figure S3. Distribution of G-quadruplex sequences in λ-phage DNA. The analysis was carried out by using the G4 hunter algorithm of the DNA analyser program package (https://bioinformatics.ibp.cz/#/analyse/quadruplex).

Supplementary Video. Time-dependent force *versus* extension data collection of a single λ-phage DNA molecule stretched and relaxed in consecutive mechanical cycles with progressively changing maximal stretch length. 320 nM TMPyP, 1 M NaCl, 20 μm/s pulling rate. The video was generated by using a user-developed Python script based on the experimentally obtained data points. In the video the data points appear at the realistic time scale.
